# Spatial and Temporal Dynamics and Value of Nature-Based Recreation, Estimated via Social Media

**DOI:** 10.1371/journal.pone.0162372

**Published:** 2016-09-09

**Authors:** Laura J. Sonter, Keri B. Watson, Spencer A. Wood, Taylor H. Ricketts

**Affiliations:** 1 The Gund Institute for Ecological Economics, University of Vermont, Burlington, VT 05405, United States of America; 2 Rubenstein School of Environment and Natural Resources, University of Vermont, Burlington, VT 05405, United States of America; 3 The Natural Capital Project, Woods Institute for the Environment, Stanford University, Stanford, CA 94305, United States of America; 4 School of Environmental and Forest Sciences, University of Washington, Seattle, WA 98195, United States of America; University of the Chinese Academy of Sciences, CHINA

## Abstract

Conserved lands provide multiple ecosystem services, including opportunities for nature-based recreation. Managing this service requires understanding the landscape attributes underpinning its provision, and how changes in land management affect its contribution to human wellbeing over time. However, evidence from both spatially explicit and temporally dynamic analyses is scarce, often due to data limitations. In this study, we investigated nature-based recreation within conserved lands in Vermont, USA. We used geotagged photographs uploaded to the photo-sharing website Flickr to quantify visits by in-state and out-of-state visitors, and we multiplied visits by mean trip expenditures to show that conserved lands contributed US $1.8 billion (US $0.18–20.2 at 95% confidence) to Vermont’s tourism industry between 2007 and 2014. We found eight landscape attributes explained the pattern of visits to conserved lands; visits were higher in larger conserved lands, with less forest cover, greater trail density and more opportunities for snow sports. Some of these attributes differed from those found in other locations, but all aligned with our understanding of recreation in Vermont. We also found that using temporally static models to inform conservation decisions may have perverse outcomes for nature-based recreation. For example, static models suggest conserved land with less forest cover receive more visits, but temporally dynamic models suggest clearing forests decreases, rather than increases, visits to these sites. Our results illustrate the importance of understanding both the spatial and temporal dynamics of ecosystem services for conservation decision-making.

## Introduction

Conserved lands provide valuable ecosystem services to billions of people worldwide. Nature-based recreation alone contributes to human physical, mental and cultural wellbeing [1] and generates more than US $600 billion annually for the global economy [2]. This value far exceeds contemporary conservation expenditures [3], yet mounting pressures from land use and climate changes continue to threaten conserved lands, their biodiversity [4] and contribution to human wellbeing [5]. In recognition, conservation scientists increasingly quantify ecosystem services provided by conserved lands and use this value to support management and spending decisions [6]. Compiling the evidence needed for this task requires understanding both the spatially-explicit landscape attributes underpinning ecosystem services, and how changes in land use and management affect their contribution to human wellbeing over time [7, 8]. Despite this, current empirical evidence for the ecosystem service of nature-based recreation is scarce—only 23% of studies are spatially explicit and 17% are multi-temporal [9].

A wide range of landscape attributes underpin nature-based recreation. For example, past studies show visitor use depends on natural features of conserved lands and their surrounding environment (e.g. biodiversity, forest cover, water quality) and built capital providing people access to these recreational sites (e.g. roads, camp facilities) [10–13]. Visitation rates are also affected by the spatial distribution of these landscape attributes [14, 15], and their value depends on the characteristics of human beneficiaries, their demand for recreation, and preferences for different recreational activities [9]. As a result, the most important landscape attributes for enhancing nature-based recreation often differ between sites and studies. More evidence is needed to obtain a general understanding of how landscape attributes and human beneficiaries affect nature-based recreation—to aid conservation decisions in information-limited contexts.

Maximizing nature-based recreation also requires understanding how visitation rates respond to changes in landscape attributes over time [16]. However, these dynamics are often inferred from static relationships (i.e. studying variability in space) rather than quantified from time series data. For example, Keeler et al. [17] found that water clarity explains the spatial distribution of visits to lakes across Iowa and Minnesota and extrapolated this relationship to predict future visitation under scenarios of improved water clarity. Such extrapolation assumes human preferences for landscape attributes remain constant over time, which is unlikely true for many cultural ecosystem services [18]. Visits to conserved lands may be initially motivated by opportunities to view species, while subsequent visits may be motivated by past site experiences. Such space-for-time substitutions also ignore interactions among sites that produce patterns that emerge over time. For example, reducing ecosystem services in one place indirectly can affect its supply or use elsewhere; relative (rather than absolute) water quality may explain visits to lakes, thus changes in water quality in one lake may redistribute (rather than increase) recreational visits across the landscape. Failing to understand these temporal dynamics may have perverse outcomes for conservation investments and human wellbeing.

Quantifying spatial and temporal dynamics of socio-ecological systems—and specifically the ecosystem services provided by conserved lands and their contribution to human wellbeing—has been limited by data availability. This is particularly true for studies seeking to quantify impacts on nature-based recreation and other forms of cultural services, which require time-consuming and expensive survey data [18]. Over the past 5 years, social media datasets, such as geotagged photographs uploaded to photo sharing websites (e.g. Flickr), have been successfully used to predict visits to recreation sites and to indicate human preferences and decision-making processes in locations with sparse empirical data [17, 19]. Other forms of social media data have also been useful indicators in similar ways [20, 21]. To date, social media data have not been used to quantify changes in recreation over large spatial and temporal scales.

In this study, we investigate nature-based recreation within conserved lands in the state of Vermont, USA. We define conserved lands as areas legally protected for the purpose of environmental conservation, and address four specific questions:

1. Can photographs uploaded to Flickr be used to indicate visits to conserved lands?
2. Which landscape attributes explain the spatial distribution of visits to conserved lands?
3. What is the value of conserved lands for the state tourism industry?
4. Do changes in landscape attributes explain changes in the spatial distribution of visits over time?

## Materials and Methods

### Study region

Conserved lands in Vermont cover approximately 5,800 km^2^ (25% of the state) and are managed by multiple entities, including government agencies (federal, state, and local), non-government organizations and local landowners. These conserved lands consist of forests interspersed throughout a semi-natural, rural landscape, and popular recreation activities for both in- and out-of-state visitors include swimming, camping, hiking, hunting and fishing, fall foliage viewing, and snow sports (including alpine and nordic skiing, snowboarding, and snowshoeing) [22]. In combination, these activities make a significant contribution to the Vermont economy—for example, annual forest recreation (within and beyond conserved lands) was recently valued at US $1.9 billion [23]. However, the proportion of this monetary value provided by conserved lands is currently unknown, as are the impacts of recent investments and changing demands on visitation rates. Investments in new infrastructure have been made to improve recreational site quality (~US $10 million investment in Vermont State Park facilities) and demand for recreation activities has changed over time (trail-based activities have increased, while hunting has decreased) [22].

### Data sources

**Fig 1:**
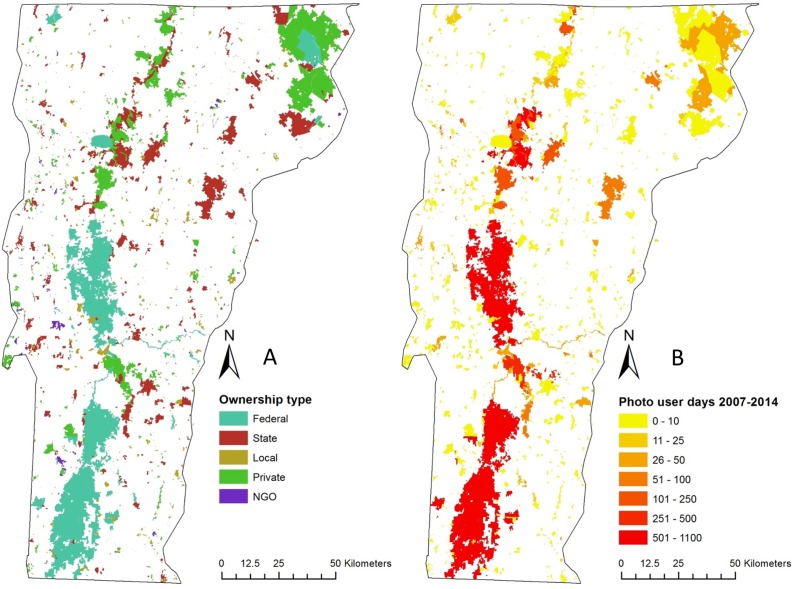
Conserved lands in Vermont. Spatial distribution of conserved lands by (A) ownership type and (B) photo user days (PUD; see description of this indicator in “Data Sources”) between 2007 and 2014.

We obtained the conserved lands map made available online by The Nature Conservancy, which classifies parcels of conserved land by their ownership type (Fig 1A) and we aggregated parcels into meaningful entities based on site name (n = 998). Geotagged photographs uploaded to the photo sharing website Flickr (www.flickr.com) were obtained for years 2007–2014 and we counted the number of individual users per day who uploaded at least one photograph within each parcel of conserved land. This metric is referred to as “photo user days” (PUD) [19] (Fig 1B). We also obtained information on the proportion of PUD by in-state and out-of-state users, based on the home locations stated by Flickr users in their account profiles.

To validate the use of PUD to indicate visits to conserved land, we obtained survey data on visitation rates—i.e. “survey user days” (SUD)—to Vermont’s state parks (n = 39) from Vermont’s Department of Forests, Parks and Recreation. SUD represent visitation during summer months (July–September) and we obtained data for years 2007–2014. We also disaggregated SUD to compare camping vs. day use visits, and in- vs. out-of-state visitors. Subsets were not mutually exclusive—visits by in-state visitors include camping and day use visits.

To explain the spatial distribution of visits to conserved lands, we obtained data on 10 potentially significant landscape attributes: size, land ownership type (private, federal, state, local and non-government organizations), land cover in 2011 (including forests, water, and developed land), recreational opportunities (for swimming, camping, and snow sports), slope, distance to towns, trail density, surrounding population, surrounding conserved lands, and surrounding road density. Table 1 describes static landscape attributes, their data sources and summary statistics for different subsets of conserved lands. Dynamic landscape attributes included changes in land cover (forest, water, developed) between years 2006 and 2011 [24]. These landscape attributes were chosen based on their importance in explaining nature-based recreation in other locations [10–15] and because they represent our knowledge of recreational preferences and activities in Vermont [22, 25, 26]. It was not our intention to comprehensively capture all landscape attributes explaining nature-based recreation, but instead to understand the relative importance of tested attributes and investigate how they differ over time and between visitors groups. All spatial data were projected to the Vermont State Plane Coordinate System, based on North American Datum (NAD) 1983.

**Table 1:** Landscape attributes of conserved lands in Vermont.

Landscape attributes		Conserved lands (PUD)	State parks (PUD, SUD)	Data source
**Size** (ha) of conserved land		Count: 998 (421)	Count: 61(34)	Conserved lands map provided by The Nature Conservancy (TNC)
		Mean: 577 (1279)	Mean: 495 (498)	
		Median: 31(78)	Median: 99 (105)	
		Sum: 577,100 (538,500)	Sum: 19,900 (18,700)	
**Ownership type** (dummy variable). Types include:				
	Private, e.g. conservation easements	Count: 174 (86)	-	
	Federal, e.g. national forest	Count: 22 (17)	-	
	State, e.g. state park	Count: 430 (183)	Count: 61 (34)	
	Local, e.g. town park	Count: 317 (113)	-	
	Non-government, e.g. TNC	Count: 53 (22)	-	
**Land cover in 2011** (% land cover within conserved lands). Categories include:				National Land Cover Database (NLCD) http://www.mrlc.gov/finddata.php
	Forest cover	Mean: 73 (73)	Mean: 73 (76)	
		Median: 89 (87)	Median: 75 (85)	
	Water cover	Mean: 10 (9)	Mean: 11 (9)	
		Median: 1.4 (2)	Median: 2.4 (3)	
	Developed cover	Mean: 10 (12)	Mean: 12 (11)	
		Median: 0.4 (2)	Median: 0.2 (2)	
**Recreation opportunities** (count of opportunities). Opportunities include:				Vermont National Resource Atlas http://anrmaps.vermont.gov/websites/anra/
	Swimming	Count: 50 (41)	Count: 21 (20)	
	Camping	Count: 111 (92)	Count: 33 (30)	
	Snow sports	Count: 38 (31)	Count: 3 (2)	
**Slope** (average slope [degrees] within conserved land)		Mean: 7.3 (7.7)	Mean: 7.2 (7.8)	Vermont Center for Geographic Information http://vcgi.vermont.gov/warehouse
		Median: 6.5 (7.4)	Median: 5.4 (4.2)	
		Min: 0 (0)	Min: 0 (0)	
		Max: 35.1 (26.6)	Max: 18.7 (18.7)	
**Distance to towns** (Euclidian distance [km] from conserved land to nearest town.		Mean: 37.5 (33.1)	Mean: 38.2 (37.5)	
		Median: 33.7 (30.3)	Median: 32.7 (32.3)	
		Min: 0 (0)	Min: 5.4 (5.4)	
		Max: 98.1 (98.1)	Max: 85.5 (85.5)	
**Trail density** (trail length [km] per ha of conserved land. Includes trails used for hiking, biking, ATV, horse-riding; forest service and private roads; and utility corridors).		Mean: 0.05 (0.06)	Mean: 0.13 (0.15)	
		Median: 0.0 (0.0)	Median: 0.11 (0.12)	
		Min: 0 (0)	Min: 0 (0)	
		Max: 0.6 (0.8)	Max: 0.76 (0.75)	
**Surrounding conserved land density** (area of conserved lands [ha] per ha of land within 25 km of conserved land)		Mean: 0.005 (0.005)	Mean: 0.005 (0.006)	
		Median: 0.005 (0.005)	Median: 0.005 (0.005)	
		Min: 0.001 (0.001)	Min: 0.001 (0.001)	
		Max: 0.017 (0.017)	Max: 0.014 (0.013)	
**Surrounding population** (mean population per km^2^ within 25 km of conserved land)		Mean: 34.5 (40.7)	Mean:32.3 (36.8)	
		Median: 23.2 (26.4)	Median: 22.0 (25.1)	
		Min: 2.8 (2.8)	Min: 2.8 (5.7)	
		Max: 162.4 (154.8)	Max: 162.4 (137.1)	
**Surrounding road density** (road length [km] per ha of land within 25 km of conserved land)		Mean: 0.2 (0.2)	Mean: 0.2 (0.2)	
		Median: 0.2 (0.2)	Median: 0.4 (0.5)	
		Min: 0.1 (0.1)	Min: 0.1 (0.1)	
		Max: 0.3 (0.3)	Max: 0.2 (0.2)	

Data sources and summary statistics for each landscape attribute for all conserved lands, and for state parks (i.e. a subset of all conserved lands). Values in parentheses represent conserved lands with >0 visit, as indicated by photo user days (PUD) and survey user days (SUD; state parks only).

To quantify the monetary value of conserved lands to Vermont’s tourism industry, we obtained data on average trip expenditures. We used indicators from the 2011 Benchmark Study of the Economic Impact of Visitor Spending on the Vermont Economy [27]. This report was prepared for the Vermont Department of Marketing and Tourism, and relies on the broadly applied IMPAN input-output model, previously published survey results, and tax receipts to estimate the economic impact of tourism on the Vermont economy. We obtained estimates of trip expenditures by in- and out-of-state (including US and international) visitors and divided these values by mean trip lengths (1.1 and 2.7 days respectively) to derive expenditures per user day for in- (US $82) and out-of-state (US $59) visitors. These values include direct expenditures only; they do not incorporate impacts on tax revenue, employment, or economic ripple effects on other sectors [28, 29] and are thus conservative estimates of the total impact of nature-based recreation on the Vermont economy.

### Statistical analysis

Statistical analyses were performed in R v.3.3.0 [30]. Linear regression was used to model the relationship between SUD (response variable) and PUD (explanatory variable) across all state parks. For each state park, we subset PUD to the same months as SUD (i.e. summer months), aggregated SUD and PUD between 2007 and 2014, excluded state parks with PUD = 0 (resulting in n = 34), and log-transformed SUD and PUD to meet assumptions of normality and homoscedasticity. We also used linear regression to model the relationship between SUD and PUD for camping vs. day-use visits, and by in- vs. out-of-state visitors separately. Resulting regression models were used to predict visits to all conserved lands in Vermont, assuming state parks were representative of other conserved lands. When PUD home locations were known, corresponding regression models were used (10% of PUD were in-state users, 25% were out-of-state); when home locations were unknown we used the pooled regression model (65% of PUD were unknown).

Multiple linear regression was used to model relationships between PUD (response variable) and landscape attributes (explanatory variables) across all conserved lands. For each parcel of conserved land, we aggregated PUD between 2007 and 2014, excluded conserved lands with PUD = 0 (resulting in n = 421), and log-transformed PUD to meet assumptions of normality and homoscedasticity. Modelled landscape attributes were not correlated (Pearson coefficient >0.7); however, some untested attributes were. For example, opportunities for boating and picnicking were correlated with opportunities for swimming, and opportunities for hiking were correlated with opportunities for camping. We also used multiple linear regression to model subsets of PUD (in- vs. out-of-state visitors). Stepwise model simplification was used to find the best model fit and we quantified the relative importance (i.e. the proportion of model variation explained by each coefficient) of each significant landscape attribute.

To quantify the value of conserved lands to Vermont’s tourism industry, we multiplied predicted visits to conserved lands by mean trip expenditures for in- (US $82) and out-of-state visitors (US $59). When visitor type was known, we multiplied visits by corresponding trip expenditures; when visitor type was unknown we multiplied visits by out-of-state trip expenditures, to be conservative.

To investigate the response in visitation rates to changes in landscape attributes over time, PUD were subset into two 4-year time periods (2007–2010 and 2011–2014). Relationships between PUD and SUD across state parks did not differ between time periods (including year as a fixed effect did not improve model fit; n = 68, F = 0.961, p>0.10). To determine differences in visits between the two 4-year time periods we used a paired t-test applied to all conserved land with PUD >0 in at least 1 time periods (n = 421). We then used multiple linear regression to model relationships between a change in PUD (response variable) and landscape attributes (including both static [Table 1] and dynamic [i.e. land cover change] attributes) as explanatory variables.

## Results

### Do Flickr photographs indicate visits to conserved lands?

**Fig 2:**
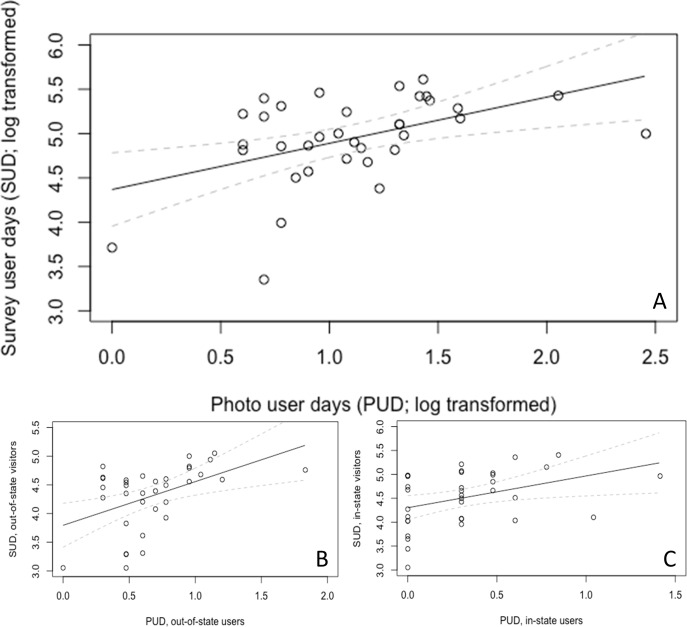
Relationships between photo user days (PUD) and survey user days (SUD) within Vermont state parks between 2007 and 2014. Graphs show log-transformed data with corresponding linear regression line and 95% confidence intervals. Panels show: (A) PUD vs. SUD; (B) PUD by in-state users vs. SUD by in-state visitors; (C) PUD by out-of-state users vs. SUD out-of-state visitors.

We found a significant and positive relationship between photos uploaded to the social media site Flickr (indicated by PUD) and survey visitation rates (indicated by SUD) within Vermont state parks (F = 9.33, p<0.01, R^2^ = 0.22, n = 34; Fig 2A; Table 2). Relationships between PUD and SUD were also significant for in- (F = 6.04, p<0.05; Fig 2B) and out-of-state (F = 9.38, p<0.01; Fig 2C) visitors, although model coefficients differed (Table 2). Each PUD by in-state Flickr users represented more visits than each PUD by out-of-state users. As expected, relationships between different subsets of PUD and SUD (e.g. PUD by in-state users, SUD by out-of-state visitors) were not significant (Table 2). Extrapolating significant relationships from state parks, we predicted conserved lands received 29.1 million visits (2.8–134.9 million at 95% confidence) between 2007 and 2014 (~3.6 million visits each year), including 5.1 million visits by in-state visitors, 6.3 million by out-of-state visitors, and 17.7 million by visitors from an unknown location.

**Table 2:** Linear regression models quantifying relationships between survey user days (SUD; log transformed) and photo user days (PUD; log transformed) within Vermont state parks.

*Photo user days (PUD)*	*Survey user days (SUD)*				
	All SUD	SUD, in-state visitors	SUD, out-of-state visitors	SUD, camping visits	SUD, day use visits
All PUD	0.52**	0.31	0.59**	0.78	0.95*
PUD, by in-state users	0.60*	0.66*	0.21	-1.55	1.43*
PUD, by out-of-state users	0.52*	0.15	0.76**	1.24	0.72

Results from 15 different models are shown, all with n = 34. Explanatory variables are shown in rows and include: PUD by all users, PUD by in-state users, and PUD by out-of-state users. Response variables are shown in columns and include: SUD by all visitors, SUD by in-state visitors, SUD by out-of-state visitors, SUD for camping visits, and SUD for day use visits. Note: that SUD subsets are not mutually exclusive—i.e. visits by in- visitors include both camping and day-use visits. For each model, the table shows model coefficients. Stars denote significance

‘**’ significant at 0.01

‘*’ significant at 0.05

### Which landscape attributes explain visits to conserved lands?

Eight landscape attributes explained 43% of the variation in visits to conserved lands as measured by PUD (F = 30.86; n = 421; Table 3). Size of conserved lands was the most important attribute, with larger conserved lands receiving more visits (t = 8.30, p<0.001). Model coefficients also suggest visits were significantly higher (p<0.05) in conserved lands with less forest cover, steeper slopes, greater trail density, more opportunities for snow sports and swimming, and greater surrounding population. Land ownership types of state, local, private and non-government also influenced the spatial distribution of visits. Non-significant landscape attributes were land cover categories of water and developed land, distance to towns, surrounding conserved land density and surrounding road density (S1 Table).

**Table 3:** Multiple linear regression models quantifying relationships between visits to conserved land, as indicated by photo user days (PUD; log transformed), and landscape attributes.

*Landscape attributes*	Photo user days (PUD)			PUD, by in-state users			PUD, by out-of-state users		
	2007–2014	2007–2010	2011–2014	2007–2014	2007–2010	2011–2014	2007–2014	2007–2010	2011–2014
Size	8.2e^-2^***	7.6e^-2^***	8.7e^-2^***	4.2e^-2^***	3.0e^-2^***	2.3e^-2^***	6.5e^-2^***	4.7e^-2^***	5.1e^-2^***
Ownership: private	-3.0e^-1^**	-2.9e^-1^***					-2.3e^-1^**	-1.3e^-1^*	-2.0e^-1^**
Ownership: state	-2.9e^-1^**	-2.6e^-1^**					-2.5e^-1^**	-1.3e^-1.^	-2.3e^-1^***
Ownership: non-government	-4.4e^-1^***	-4.2e^-1^***					-3.1e^-1^**	-1.8e^-1^*	-2.8e^-1^**
Ownership: local	-3.5e^-1^***	-3.2e^-1^**					-3.2e^-1^***	-2.0e^-1^**	-2.9e^-1^***
Land cover in 2011: forest	-4.4e^-3^***	-4.0e^-3^**	-3.8e^-3^***	-2.7e^-3^***	-2.3e^-3^***		-3.0e^-3^***	-2.9e^-3^***	-2.1e^-3^**
Land cover in 2011: water			2.4e^-3^*				1.0e^-3.^		1.8e^-3^*
Opportunities for swimming	2.4e^-1^***	2.1e^-1^***	2.0e^-1^***				1.8e^-1^***	1.7e^-1^***	1.0e^-1^**
Opportunities for snow sports	3.0e^-1^***	2.9e^-1^***	3.1e^-1^***	2.9e^-1^***	2.2e^-1^***	2.7e^-1^***	2.9e^-1^***	2.4e^-1^***	2.6e^-1^***
Slope	2.3e^-2^***	2.3e^-2^***	1.7e^-2^***	1.3e^-2^***	1.3e^-2^***		1.9e^-2^***	1.3e^-2^***	1.4e^-2^***
Trail density	5.9e^-1^***	5.1e^-1^***	5.8e^-1^***	2.9e^-1^**	2.5e^-1^**	2.0e^-1^*	4.8e^-1^***	3.9e^-1^***	3.8e^-1^***
Surrounding population density	1.6e^-3^**	1.2e^-3^*	1.5e^-3^**	2.2e^-3^***	1.3e^-3^***	1.4e^-3^***			
**F-statistic**	30.8***	26.3***	33.7***	35.5***	30.7***	44.8***	29.0***	27.4***	25.3***
**Adjusted R**^**2**^	**0.43**	**0.39**	**0.38**	**0.33**	**0.30**	**0.29**	**0.42**	**0.38**	**0.39**

Results from nine models, all with n = 421, are shown in columns. Response variables are PUD by all users, PUD by in-state users, and PUD by out-of-state users, for three time periods: 2007–2014, 2007–2010, and 2011–2014. Values are model coefficients for significant landscape attributes. Stars denote significance

‘***’ significant at 0.001

‘**’ significant at 0.01

‘*’ significant at 0.05

‘.’ significant at 0.1

Saturated models are shown in S1 Table.

Significant landscape attributes also varied for different subsets of visitors (Table 3). For in-state visitors, the most important landscape attribute was opportunities for snow sports (t = 7.80, p<0.001), which accounted for 42% of explained model variation. For out-of-state visitors, the most important attribute was size of conserved land (t = 7.51, p<0.001), which accounted for 30% of variation. Further, land ownership, water cover and opportunities for swimming were significantly correlated with visits by in-state visitors but not with visits by out-of-state visitors, and surrounding population density was correlated with visits by in-state visitors but not by out-of-state visitors (Table 2).

### What is the value of conserved lands to VT tourism industry?

Multiplying predicted visits by mean trip expenditures for in- (US $82) and out-of-state visitors (US $59) (see methods), suggests conserved lands contributed US $1.8 billion (US $0.18–20.2 billion at 95% confidence intervals) to Vermont’s tourism industry between 2007 and 2014 (US $230.2 million per year).

### Do changes in landscape attributes explain changes in visits?

Visits to conserved lands increased significantly between the two 4-year time periods (t = 1.88, p = 0.06). Three landscape attributes significantly explained changes visits to conserved lands over time (F = 5.03, p<0.05; Table 4), although model explanatory power was low (R^2^ = 0.03; n = 421). Opportunities for snow sports was the most important attribute (p<0.01), while distance to towns and slope were also significant (p<0.05). Model coefficients suggest visits increased in conserved lands with greater opportunities for snow sports, flatter slopes and greater proximity to towns. However, landscape attributes explaining changes in visits also differed between in- and out-of-state visitors (Table 4). Visits to conserved lands with less forest loss and greater proximity to towns increased in visits by in-state visitors, but not by out-of-state visitors. Opportunities for snow sports and swimming, meanwhile, explained changes in visits by out-of-state visitors, but not by in-state visitors.

**Table 4:** Multiple linear regression models quantifying relationships between change in visits to conserved land (as indicated by a change in photo user days [PUD] between two time periods [2007–2010 and 2011–2014] and landscape attributes (including static and dynamic attributes).

*Static and dynamic landscape attributes*	Change in photo user days (ΔPUD)	ΔPUD, by in-state users	ΔPUD, by out-of-state users
Slope	-0.18.	-5.30e^-2^***	-0.06*
Opportunities for snow sports	2.57**		-0.94*
Opportunities for swimming			-2.02***
Distance to towns	-3.50^e-5^*	-7.80e^-6^*	
Land cover change: forest loss (2006–2011)		-4.28***	
**F-statistic**	5.03***	**15.69** ***	14.13***
**Adjusted R**^**2**^	**0.03**	**0.09**	**0.09**

Results from three different models, all with n = 421, are shown in columns. Response variables were PUD by all users, PUD by in-state users, and PUD by out-of-state users. Values are the model coefficients for all significant landscape attributes. Stars denote significance

‘***’ significant at 0.001

‘**’ significant at 0.01

‘*’ significant at 0.05

Saturated models are shown in S2 Table.

Many of the landscape attributes explaining variation in visits across sites differed from those explaining changes in visits to these sites over time. For example, while visits increased in conserved lands with greater proximity to towns (Table 4), this attribute did not significantly explain variation over space (Table 3). Differences were also evident for subsets of visitors. For example, while visits by in-state visitors increased in conserved lands with less forest loss (Table 4), visits were greater in conserved lands with less forest cover (Table 3); and while visits by out-of-state visitors decreased in conserved land with more opportunities for snow sports and swimming (Table 4), visits were greater in conserved lands with more of these opportunities (Table 3).

## Discussion

Vermont’s conserved lands provide opportunities for nature-based recreation by in- and out-of-state visitors and contributed an estimated US $1.8 billion to the tourism industry between 2007 and 2014. Geotagged photographs uploaded to Flickr can be used to predict visits to conserved lands in Vermont, but relationships between PUD and SUD differed for in- and out-of-state visitors. Eight landscape attributes significantly explained the spatial distribution of visits to conserved lands. Some attributes differed from those previously reported in other locations, but all aligned with our understanding of recreation preferences and activities by in-state and out-of-state visitors in Vermont. Visits to conserved lands changed over time, but models predicting differences among sites at a given time do not predict well changes in visits to these sites over time—thus, the common approach of space-for-time substitution may not be valid here. This finding illustrates the importance of understanding and using both the spatial and temporal dynamics of ecosystem services to inform land management and conservation decisions.

### Using social media data to estimate nature-based recreation

Geotagged photographs uploaded to the photo sharing website Flickr were significantly and positively correlated with survey visits to Vermont’s state parks (Table 2; Fig 2). This suggests that social media data can be used to predict visits to data-sparse recreational sites. Our models suggest conserved lands received an estimated 29.1 million visits between 2007 and 2014: approximately 3.6 million visits per year. To make these projections, we assumed state parks were representative of all conserved lands, which was reasonable given that annual PUD followed a similar trend for state parks and all conserved lands (S1 Fig) and the landscape attributes of state parks were not starkly different from other conserved lands, although state parks were smaller in size (Table 1). Comparisons with previously published statistics on nature-based recreation in Vermont suggest our user-day projections are conservative, yet within the correct order of magnitude. For example, Vermont attracts 9–11 million out-of-state visitors each year with an average trip length of 8 nights, and of these ~10–30% are motivated by nature-based recreation opportunities, such as wildlife viewing, hiking and kayaking [22, 26]. This suggests visits by out-of-state visitors contribute roughly 7.2–26.4 million user days each year. Similarly, data published by the US Forest Service suggests that visits (defined as the entry of one person into the site for an unspecified period of time) to Green Mount National Forest alone—Vermont’s largest conserved land and only national forest—equaled ~2.4 million annually [31].

Despite finding a significant relationship between PUD and SUD, our model had relatively low explanatory power (R^2^ = 0.22) compared to past studies that use PUD to indicate nature-based recreation (e.g. Wood *et al*. [19] report R^2^ = 0.38; Keeler *et al*. [17] report R^2^ = 0.65). Two factors may explain these differences. First, our survey dataset likely had less variation in the explanatory variable since we examined only one type of conserved land (state parks), whereas Wood et al. included a wider range of both cultural attractions (e.g. Disneyland) and natural places (e.g. Yellowstone Park). Second, visitor types and recreational activities likely differed between studies, which we found to significantly influence relationships between PUD and SUD (Table 2; Fig 2B and 2C). Therefore, although PUD can be a useful indicator, social media data should be compared to, and wherever possible used in tandem with survey data to quantify potential sources of uncertainties in predictions. Our results also suggest future analyses should strive to incorporate information on specific visitor groups (e.g. in- vs. out-of-state visitors) and recreational activities (e.g. camping vs. day-use visits), since these factors altered relationships between PUD and SUD in Vermont (Table 2; Fig 2).

### Landscape attributes explaining visits to conserved lands

Eight landscape attributes significantly explained the spatial distribution of visits (as indicated by PUD) to Vermont’s conserved lands (Table 3). These attributes included natural landscape features that underpin ecosystem services and other forms of built capital that provide or enhance access to recreational sites. We found the size of conserved lands was the most significant attribute, followed by opportunities for snow sports and swimming, land ownership, trail density, slope, forest cover, and surrounding population. These findings align with local knowledge of recreation in Vermont, regarding human preferences and popular recreational activities, and thus further support use of social media in predicting recreational visits to conserved land. For example, trail-based activities and opportunities for swimming have been identified as important sources of recreational demand in recent years and these landscape attributes have also been identified as future conservation priorities to increase the value of conserved lands [22].

The most important landscape attributes differed between in- and out-of-state visitors (Table 3). Nearby population explained visits by in-state visitors, but had no effect on out-of-state visitors who had already traveled to visit Vermont. Similarly, opportunities for snow sports explained visits by in-state visitors, while opportunities for swimming were more important to out-of-state visitors. This finding was expected since most out-of-state visits to conserved lands in Vermont occur during the summer months, when swimming, rather than snow sports, is the desired activity [26]. In general, our results illustrate the importance of understanding how preferences for recreation activities differ between groups of recreationists when managing nature-based recreation. This conclusion is supported by many other authors. For example, Tyrväinen *et al*. [32] found that foreigners are willing to pay more for forest conservation than domestic tourists in Finland, and that incorporating this information into land management decisions improves the outcomes of projects aimed to improve the quality of recreational sites.

Many landscape attributes that explained visits to conserved lands in Vermont have also been found important elsewhere; however, two attributes opposed previous findings and thus deserve further attention here. We found visits were higher in conserved lands with less forest cover (Table 2), while other studies report visits to be higher in densely forested sites [10, 12, 33]. Many factors potentially explain this difference; perhaps the most important being that forests are not a scarce resource in Vermont—more than 80% of the landscape is currently forested. Thus, it is unlikely that this landscape attribute is unlikely attracting visitors to specific conserved lands. Additionally, many visitors to Vermont are motivated by its rural “working” landscape [26], which has lower forest cover due to being used for multiple non-forest purposes. For example, working agricultural properties conserved by the Vermont Land Trust had an average forest cover of just 55%. Other conserved lands with high visitation and low forest cover included local parks developed to enhance accessibility to swimming holes, picnic areas and sporting facilities. For example, Oakledge Park—a popular local park in Vermont’s largest city, Burlington—has a forest cover of just 8%. Therefore, our forest cover finding likely reflects the value of accessible conserved lands interspersed throughout natural forests [22] and demonstrates the need to understand attributes of the broader landscape that recreation areas exist within. In our case, if Vermont were not heavily forested, preferences for this landscape attribute would have likely differed.

We also found landscape attributes surrounding conserved lands (e.g. surrounding road density, distance to towns, surrounding conserved land density) were not important in explaining visits to conserved lands in Vermont (S1 Table), but were found important elsewhere [10, 11, 13]. This may be because conserved lands exist throughout the state and the maximum distance between any conserved land and a town is less than 100 km (Table 1), making them accessible by car within 1–2 hours. Similarly, most conserved lands have good infrastructure allowing access without being overdeveloped so as to degrade site quality. Our results also likely reflect Vermont’s tourism industry being dominated by US visitors from the New England area and Canadian visitors from the nearby province of Quebec [25, 26]. Most (>75%) of these out-of-state visitors travel in their personally-owned automobiles [34] and thus distance to towns and surrounding road density is unlikely to limit access to conserved lands.

The landscape attributes investigated in this study are a subset of all possible attributes explaining nature-based recreation within Vermont’s conserved lands. Our subset was chosen based on their importance for nature-based recreation in other studies and locations [10–15] and our knowledge of recreational preferences and activities in Vermont [22, 25, 26]. Some additional predictors may be correlated with our subset of attributes. For example, landscape aesthetics often depend on land cover and terrain [35]. Others attributes may influence recreation, such as the spatially-distributed effects of climate change [35, 36]. Thus, our results did not comprehensively assess all possible landscape attributes, but instead illustrate preferences for those in our subset, and show how these preferences differ between visitor groups over time.

### Monetary value of nature-based recreation

We estimated that conserved lands contributed US $1.8 billion to the Vermont tourism industry between 2007 and 2014 in the form of trip expenditures (~ US $230.2 million per year). Although these values were lower than those estimated in previous studies (see below), they far exceed current economic investment in conserved lands. For example, the budget allocated to maintain Vermont’s state parks in 2014 equaled US $7.9 million [37], while our estimates suggest state parks contributed US $136 million in that year (considering state parks received 1.8 million visits; 65% by in-state and 35% by out-of-state visitors). Nature-based recreation accounts for over half of the forest-based economy in Vermont, exceeding the value of logging, wood products, wood energy, maple, and paper industries combined [23]. However, without adequate investment, this ecosystem service will likely diminish under future land use and climate changes.

Our estimated value of conserved lands was less than previous estimates. For example, annual forest recreation in Vermont (including recreation within and beyond conserved lands) has been valued at US $1.9 billion [23] with state parks contributing US $75 million during the summer months [22]. There are many reasons why our estimates may underestimate the value of conserved lands. First, many out-of-state visitors may be drawn to Vermont by the natural landscape provided by conserved lands, including forest cover, but may not visit conserved lands themselves [38]. Second, mean trip expenditures used in our calculations are conservative. Outdoor recreationists have higher expenditures than average Vermont visitors [34], but general averages were used here because data were more recent. Using a value specific for outdoor recreation, our annual value of conserved lands would increase to US $431.6 million. Third, the direct expenditures used in this study represent only a small proportion of the total economic impact of visitor spending. If we assume that the same ripple effects (induced and indirect economic impacts) apply to nature-based recreation, our annual value increases to US $331.5 million.

### Predicting temporal changes in nature-based recreation

Many studies on nature-based recreation use models explaining differences in visits among sites at a given time to predict changes in visits to these sites over time. However, we found this common approach of space-for-time substitution may not always be valid, since changes in visits were explained by a different set of landscape attributes than those included in the static models (Table 3 and Table 4). Visits by in-state visitors increased over time in conserved lands with less forest cover loss, flatter slopes, greater proximity to towns and fewer opportunities for swimming (Table 4). These results oppose those expected from our static models, which, for example, would predict visits to increase in conserved lands with more forest cover loss (Table 3). In other words, in-state visitors favored sites with less forest cover, but did not respond positively to forest loss. This makes sense if low forest cover is associated with improved opportunities to view wildlife, as suggested earlier, since forest loss would also negatively impact these opportunities. Similarly perverse results were found for out-of-state visitors, which were drawn to conserved lands with fewer opportunities for swimming (Table 4), whereas static models would have suggested visits to decrease in these areas (Table 3). Our results illustrate the risks of using static models to predict changes in nature-based recreation over time, and to quantify expected benefits of conservation and management interventions. To minimize such risks, future research should examine landscape attributes that explain both the spatial distribution and temporal changes in nature-based recreation.

## Conclusions

Managing and enhancing nature-based recreation requires understanding both the spatially-explicit landscape attributes underpinning this ecosystem service and how changes in land use and management affect its contribution to human wellbeing over time. However, large-scale analyses are scarce, often due to the time required to quantify trends in visitation rates using traditional survey methods [9]. In this study, we found social media data can be used to indicate visits to conserved lands, although a high degree of variability was due to whether visits were by in- or out-of-state visitors. We estimated that Vermont’s conserved lands received 29.1 million visits, and contributed US $1.8 billion to the state tourism industry in the form of trip expenditures, between 2007 and 2014. We identified eight landscape attributes that explained the spatial distribution of visits to conserved lands and these results aligned with our local knowledge of recreation preferences and activities by in-state and out-of-state visitors; however, some of our findings differed from those reported from studies conducted in other locations, demonstrating the importance of understanding local context. We found that spatial relationships between visits and landscape attributes alone cannot always be extrapolated to predict temporal changes in visits to these sites. In this study, a distinct set of landscape attributes explained changes in visits over time, and these opposed relationships suggested by temporally static models. Further research should quantify the dynamic set of landscape attributes that better explain changes in nature-based recreation over time. Incorrect information could have perverse outcomes for conservation and human wellbeing.

## Supporting Information

S1 Fig Annual photo user days within conserved lands between 2007 and 2014. PUD for state parks during the summer months (black series) and PUD for all conserved lands throughout the entire calendar year (grey series).
(DOCX)

S1 Table Saturated multiple linear regression models quantifying relationships between visits to conserved land, as indicated by photo user days (PUD; log transformed), and landscape attributes. Results from nine different models, all with n = 421, are shown in columns. Model response variables included all PUD, PUD by in-state users, and PUD by out-of-state users; for each of three time periods: 2007–2014, 2007–2010, and 2011–2014. The table shows model coefficients for all landscape attributes for each model. Stars denote significance: 0 ‘***’ 0.001 ‘**’ 0.01 ‘*’ 0.05 ‘.’ 0.1 ‘ ‘ 1.
(DOCX)

S2 Table Saturated multiple linear regression models quantifying relationships between change in visits to conserved land (as indicated by a change in photo user days [PUD] between two time periods [2007–2010 and 2011–2014] and landscape attributes (including static and dynamic attributes). Results from three different models, all with n = 421, are shown in columns. Model response variables included: all PUD, PUD by in-state visitors only, and PUD by out-of-state visitors only. For each model, the table shows model coefficients for all tested landscape attributes. Stars denote significance: 0 ‘***’ 0.001 ‘**’ 0.01 ‘*’ 0.05 ‘.’ 0.1 ‘ ‘ 1.
(DOCX)
