# Supplementary material for: Spatial and Temporal Dynamics and Value of Nature-Based Recreation, Estimated via Social Media
Source: PLoS One. 2016 Sep 9;11(9):e0162372. doi: 10.1371/journal.pone.0162372 (PMC5017630; doi:10.1371/journal.pone.0162372)
Supplement: S1 Fig — PUD for state parks during the summer months (black series) and PUD for all conserved lands throughout the entire calendar year (grey series). (DOCX) [file pone.0162372.s001.docx]

**S1 Fig. Annual photo user days (PUD) within conserved lands between 2007 and 2014.** Figure shows PUD for state parks during the summer months (black series) and PUD for all conserved lands throughout the calendar year (grey series).
