# Supplementary material for: Spatial and Temporal Dynamics and Value of Nature-Based Recreation, Estimated via Social Media
Source: PLoS One. 2016 Sep 9;11(9):e0162372. doi: 10.1371/journal.pone.0162372 (PMC5017630; doi:10.1371/journal.pone.0162372)
Supplement: S1 Table — Results from nine different models, all with n = 421, are shown in columns. Model response variables included all PUD, PUD by in-state users, and PUD by out-of-state users; for each of three time periods: 2007–2014, 2007–2010, and 2011–2014. The table shows model coefficients for all landscape attributes for each model. Stars denote significance: 0 ‘***’ 0.001 ‘**’ 0.01 ‘*’ 0.05 ‘.’ 0.1 ‘ ‘ 1. (DOCX) [file pone.0162372.s002.docx]

**S1 Table. Saturated multiple linear regression models quantifying relationships between visits to conserved land, as indicated by photo user days (PUD; log transformed), and landscape attributes.**

| **Landscape attributes** | **Photo user days (PUD)** | | | | | **PUD by in-state users** | | | | **PUD by out-of-state users** | | | |
| --- | --- | --- | --- | --- | --- | --- | --- | --- | --- | --- | --- | --- | --- |
|  | **2007–2014** | **2007–2010** | **2011–2014** | | **2007–2014** | | **2007–2010** | **2011–2014** | **2007–2014** | | **2007–2010** | **2011–2014** |  |
| Area | 8.99e^-02***^ | 8.26e^-02***^ | 8.23e^-02***^ | | 4.59e^-02***^ | | 3.53e^-02***^ | 3.04e^-02***^ | 6.90e^-02***^ | | 5.26e^-02***^ | 5.52e^-02***^ |  |
| Ownership: private | -2.71e^-01**^ | -2.75e^-01**^ | -1.48e^-01^ | | -2.32e^-02^ | | 1.55e^-02^ | -1.16e^-02^ | -2.08e^-01*^ | | -1.22e^-01^ | -1.80e^-01*^ |  |
| Ownership: state | -2.85e^-01**^ | -2.46e^-01*^ | -2.01e^-01*^ | | -2.45e^-02^ | | 3.56e^-02^ | -4.10e^-02^ | -2.35e^-01**^ | | -1.21e^-01^. | -2.23e^-01**^ |  |
| Ownership: non-government | -4.22^e-01***^ | -4.09e^-01**^ | | -2.77e^-01*^ | -7.65e^-02^ | | 7.95e^-03^ | -6.96e^-02^ | -3.04e^-01**^ | | -1.74e^-01^. | -2.73e^-01**^ |  |
| Ownership: local | -3.44e^-01***^ | -3.21e^-01**^ | -2.48e^-01*^ | | -2.06e^-02^ | | 2.64e^-02^ | -2.65e^-02^ | -3.18e^-01***^ | | -1.95e^-01**^ | -2.86e^-01***^ |  |
| Land cover in 2011: forest | -4.31e^-03**^ | -3.72e^-03**^ | -4.65e^-03**^ | | -3.21e^-03**^ | | -2.46e^-03**^ | -1.99e^-03*^ | -3.60e^-03**^ | | -2.82e^-03**^ | -3.18e^-03**^ |  |
| Land cover in 2011: water | 1.54e^-03^ | 1.31e^-03^ | 1.26e^-03^ | | 2.42e^-04^ | | 5.01e^-04^ | -3.69e^-05^ | 1.42e^-03^ | | 9.01e^-04^ | 9.21e^-04^ |  |
| Land cover in 2011: developed | -9.03e^-04^ | -1.49e^-04^ | -2.09e^-03^ | | -1.32e^-03^ | | -5.75e^-04^ | -1.43e^-03^ | -8.95e^-04^ | | -5.59e^-04^ | -1.46e^-03^ |  |
| Opportunities for swimming | 2.41e^-01***^ | 2.11e^-01***^ | 2.11e^-01***^ | | 8.05e^-02*^ | | 3.73e^-02^ | 6.29e^-02*^ | 1.90e^-01***^ | | 1.71e^-01***^ | 1.07e^-01**^ |  |
| Opportunities for snow sports | 2.93e^-01***^ | 2.92e^-01***^ | 2.99e^-01***^ | | 2.60e^-01***^ | | 2.09e^-01***^ | 2.41e^-01***^ | 2.61e^-01***^ | | 2.33e^-01***^ | 2.47e^-01***^ |  |
| Slope | 2.05e^-02***^ | 2.06e^-02***^ | 1.79e^-02***^ | | 1.27e^-02***^ | | 1.23e^-02***^ | 5.09e^-03^. | 1.80e^-02***^ | | 1.59e^-02***^ | 1.34e^-02***^ |  |
| Distance to towns | -2.09e^-07^ | 1.52e^-06^ | -1.39e^-06^ | | 1.02e^-06^ | | 1.72e^-06*^ | -7.66e^-07^ | -1.49e^-07^ | | -4.82e^-07^ | 8.32e^-07^ |  |
| Trail density | 5.89e^-01***^ | 5.26e^-01***^ | 5.57e^-01***^ | | 2.62e^-01*^ | | 2.41e^-01**^ | 1.50e^-01^. | 4.82e^-01***^ | | 3.59e^-01**^ | 3.82e^-01***^ |  |
| Surrounding conserved land density | -2.96e^+00^ | 8.75e^+00^ | -1.01e^+01^ | | 7.972e^-01^ | | 6.18e^+00^ | -4.99e^+00^ | 6.454e^-01^ | | 7.75e^-01^ | 4.45e^-01^ |  |
| Surrounding population | 1.27e^-03^. | 1.14e^-03^ | 1.05e^-03^ | | 2.33e^-03***^ | | 1.548e^-03***^ | 1.32e^-03**^ | -3.15e^-04^ | | -3.66e^-05^ | -1.35e^-04^ |  |
| Surrounding road density | 7.66e^-01^ | 2.16e^+00^. | -6.63e^-02^ | | 9.40e^-01^ | | 1.44e^+00*^ | -2.96e^-01^ | 1.47e^+00^ | | 8.59e^-01^ | 1.58e^+00^. |  |

Results from nine different models, all with n=421, are shown in columns. Model response variables included all PUD, PUD by in-state users, and PUD by out-of-state users; for each of three time periods: 2007–2014, 2007–2010, and 2011–2014. The table shows model coefficients for all landscape attributes for each model. Stars denote significance: ‘***’ significant at 0.001, ‘**’ significant at 0.01, ‘*’ significant at 0.05, ‘.’ significant at 0.1.
