# Supplementary material for: Spatial and Temporal Dynamics and Value of Nature-Based Recreation, Estimated via Social Media
Source: PLoS One. 2016 Sep 9;11(9):e0162372. doi: 10.1371/journal.pone.0162372 (PMC5017630; doi:10.1371/journal.pone.0162372)
Supplement: S2 Table — Results from three different models, all with n = 421, are shown in columns. Model response variables included: all PUD, PUD by in-state visitors only, and PUD by out-of-state visitors only. For each model, the table shows model coefficients for all tested landscape attributes. Stars denote significance: 0 ‘***’ 0.001 ‘**’ 0.01 ‘*’ 0.05 ‘.’ 0.1 ‘ ‘ 1. (DOCX) [file pone.0162372.s003.docx]

**S2 Table. Saturated multiple linear regression models quantifying relationships between change in visits to conserved land (as indicated by a change in photo user days [PUD] between two time periods [2007–2010 and 2011–2014] and landscape attributes (including static and dynamic attributes).**

| **Landscape attributes** | **Changes in photo user days (∆PUD)** | **∆PUD by in-state users** | **∆PUD by out-of-state users** |
| --- | --- | --- | --- |
| Size | 1.90e^-01^ | -8.84e^-02*^ | -6.78e^-03^ |
| Ownership: private | 3.38e^+00^. | 2.98e^-01^ | 1.94e^+00*^ |
| Ownership: state | 2.67e^+00^ | 1.40e^-01^ | 1.42e^+00^. |
| Ownership: non-government | 2.94e^+00^ | 1.10e^-01^ | 1.47e^+00^ |
| Ownership: local | 2.99e^+00^ | 1.91e^-01^ | 1.60e^+00^. |
| Land cover in 2011: forest | -2.40e^-02^ | 7.46e^-03^ | 2.21e^-03^ |
| Land cover in 2011: water | -7.17e^-03^ | -2.12e^-03^ | -2.45e^-03^ |
| Land cover in 2011: developed | -2.00e^-02^ | 3.30e^-05^ | -1.05e^-04^ |
| Land cover change: regrowth per ha conserved land (2006–2011) | 3.50e^+02^ | -2.86e^+01^ | 1.96e^+02^ |
| Land cover change: forest loss per ha conserved land (2006–2011) | -5.58e^+00^ | -3.72e^+00***^ | 7.67e^-01^ |
| Opportunities for swimming | -5.47e^-01^ | -2.96e^-01^ | -1.95e^+00***^ |
| Opportunities for snow sports | 2.56e^+00*^ | 2.17e^-01^ | -9.00e^-01^. |
| Slope | -1.14e^-01^ | -6.53e^-02***^ | -7.97e^-02*^ |
| Distance to towns | -4.06e^-05^. | -1.41e^-05**^ | 6.53e^-06^ |
| Trail density | 5.88e^-01^ | -4.97e^-01^ | 5.42e^-01^ |
| Surrounding conserved land density | 7.03e^+00^ | -4.75^e+01^ | 4.52e^+01^ |
| Surrounding population | -2.53e^-03^ | -1.29e^-03^ | -1.09e^-03^ |
| Surrounding road density | 2.89e^+00^ | -8.70e^+00^ | 5.50e^+00^ |

Results from three different models, all with n=421, are shown in columns. Model response variables included: all PUD, PUD by in-state visitors only, and PUD by out-of-state visitors only. For each model, the table shows model coefficients for all tested landscape attributes. Stars denote significance: ‘***’ significant at 0.001, ‘**’ significant at 0.01, ‘*’ significant at 0.05, ‘.’ significant at 0.1.
